# Renal Cell Carcinoma Metastasis to Anterior Orbit

**DOI:** 10.7759/cureus.15173

**Published:** 2021-05-22

**Authors:** Yong S Lee, Samaneh Davoudi, John T LiVecchi

**Affiliations:** 1 Ophthalmology, University of Central Florida College of Medicine, Orlando, USA; 2 Ophthalmology, University of Florida, Gainesville, USA

**Keywords:** orbit, renal cell carcinoma, tumor, oculoplastic surgery, metastasis

## Abstract

Renal cell carcinoma (RCC) is a rare malignancy that often metastasizes to the lung, bones, liver, and brain. Only a few cases of RCC metastasis in periocular areas have been reported in the literature. This case report describes a 70-year-old male who was presented to the University of Florida, Gainesville ophthalmology clinic with two-day symptoms of diplopia, decreased vision, and mechanical ptosis of the left eye with superior temporal mass. The patient had a history of both prostate and RCC that were in remission for five years leading to his presentation. Excisional biopsy confirmed the metastasis of RCC to the eye. The patient reported no systemic symptoms. This report also reviews existing literature on RCC metastasis to the eye and orbit. Ultimately, RCC metastasis to the eye in patients with a history of known RCC should be considered in the differential diagnosis in those presenting with an atypical mass in periocular regions.

## Introduction

Renal cell carcinoma (RCC) is a rare malignancy accounting for approximately 80-85% of renal tumors but only accounting for 2-3% of systemic tumors [1,2]. The incidence of sporadic RCC is the highest in elderly men in their 70s and 80s, with men having twice as high an incidence as women [1,2]. RCC is known to metastasize to any part of the body, with the most common sites being lung, bone, liver, and brain [1,2]. Ocular metastasis of RCC is extremely rare, with only a few cases reported in the literature. This report is of a 70-year-old male with a history of RCC presenting with RCC metastasis to the eye without other systemic manifestations of RCC while in remission for the past five years. The collection and evaluation of protected patient health information were Health Information Protection and Portability Act (HIPPA) compliant.

## Case presentation

**Figure 1:**
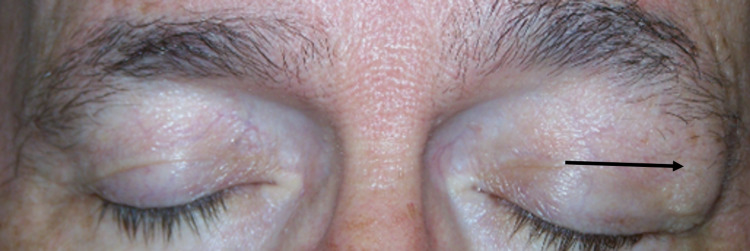
Left orbital mass The black arrow points to the left orbital mass in the superior tarsus.

A 70-year-old male presented to the ophthalmology clinic for blurry vision, diplopia, and a left upper eyelid mass. The patient stated that the blurry vision and diplopia started two days prior, and the left upper eyelid mass had been subjectively growing over the past few weeks causing the left eye to droop (Figure 1).

**Figure 2:**
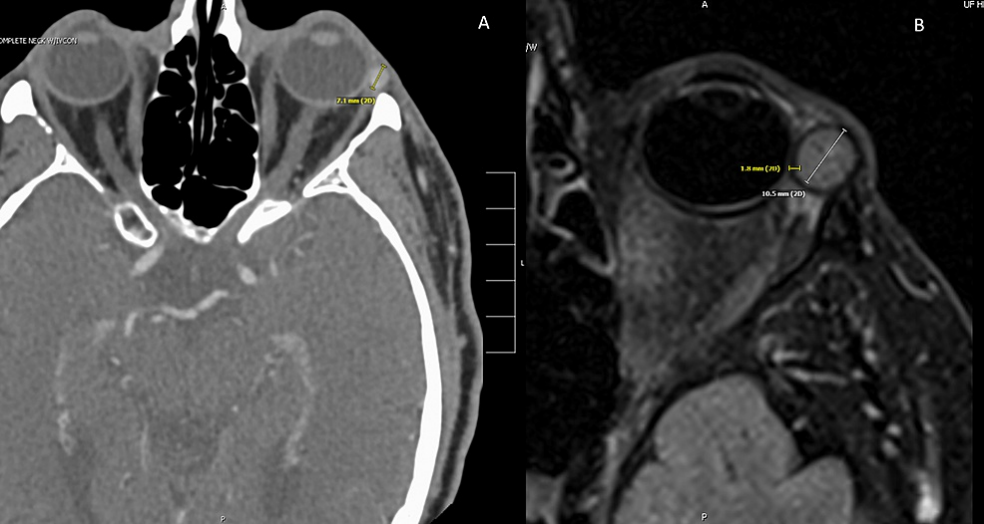
CT image (A) and MRI image (B) of left orbital mass

Past medical history was significant for RCC and prostate cancer. The patient had been in remission for the past five years from both RCC and prostate cancer. Past ocular history was significant for bilateral senile nuclear sclerosis, bilateral blepharitis of both superior and inferior tarsi, and bilateral keratoconjunctivitis sicca. The best corrected visual acuity was 20/20 in both eyes. The intraocular pressure by applanation tonometry was 12 mmHg (normal range 10-21 mmHg) in both eyes. Examination revealed mild diplopia with lateral and upward gaze. Lid examination revealed swelling of the superior tarsus and left eye ptosis. The right eye was unremarkable. The patient denied any pain associated with the mass but reported increased tearing. CT imaging from 13 months prior noted a 6-7 mm mass in the left lateral canthus of the eye (Figure 2A). MRI indicated that the same mass had grown to 11-12 mm and was well encapsulated by 1-2 mm thick fibrous capsule along the lateral orbital rim just inferior to the lacrimal gland. This mass did not involve the lacrimal gland (Figure 2B).

**Figure 3:**
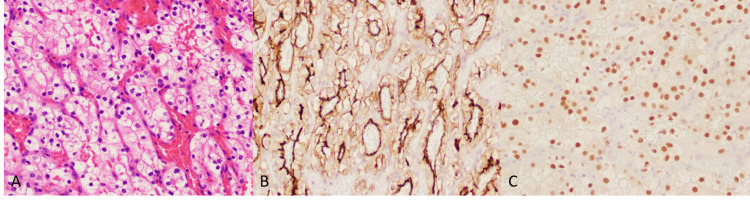
Immunohistochemistry stain of excised orbital mass (A) Hematoxylin and eosin stain at 40x, (B) CD10 stain, and (C) PAX-8 stain CD10: cluster of differentiation 10; PAX-8: paired box gene 8 stain

The patient underwent surgical excision of the mass in the left eye. A histological description of the specimen is provided in (Figure 3A). Tumor cells displayed prominent clear cell carcinoma which is the most common subtype of RCC. In addition, RCC histopathology and were strongly immunoreactive for the cluster of differentiation 10 (CD10), paired box gene 8 stain (PAX-8), and epithelial membrane antigen (Figures 3B, 3C).

PET scanning demonstrated no evidence of RCC metastasis elsewhere in the body. The patient continues to be seen clinically and has remained negative for any further recurrences of RCC.

## Discussion

Orbital and tarsal masses originating from metastasis are rare occurrences. One study showed that only 3-7% of such masses were from the metastasis of tumors, with the majority of such tumors could be attributed to tumor metastases from the breast in women and lung and prostate in men [2]. The most common presenting ocular symptoms of ophthalmic tumors are proptosis or diplopia followed by ptosis, enophthalmos, pain, and decreased visual acuity [2,3]. RCC is a rare systemic malignancy comprising 2-4% of all tumors [2,3]. RCC most commonly metastasizes to the lung, bones, liver, and brain [2]. Although RCC has the potential to metastasize to other parts of the body, ophthalmic metastases are very rare. The orbit is the most likely the site of RCC metastasis to the eye followed by the choroid, iris, and lacrimal gland [2-4]. The eyelid is a rare site of metastasis of RCC in the eye as only a few cases were reported in the literature [2,4]. 

Masses in the anterior orbit can be often overlooked as many benign conditions present as a mass in the eyelid, including but not limited to a chalazion, hidrocystoma, amyloidosis, dermal fibrosis, papilloma, and epidermoid cyst [5]. In addition, clear cell RCC, the most common subtype of RCC, presents similarly to amelanotic choroidal melanoma due to its appearance as a lightly pigmented choroidal mass [3,4,6]. Therefore, diagnosis of RCC should be suspected in patients presenting with a mass in the eyelid with a previous history of RCC, even if the patient presumes to be in remission. This was seen with the patient presented in this case and in another case where the patient was in remission of RCC for 20 years [3,4]. It has been reported that RCC metastasis to the eye can be the first presenting sign of RCC leading to a diagnosis of RCC in patients [1,2]. The clinical diagnosis of RCC is difficult without biopsy. One study showed that only 16.4% of confirmed ocular and orbital RCC metastasis are accurately diagnosed [2]. Based on these findings, if the suspicion for RCC is strong, one should proceed with a biopsy to confirm the diagnosis.

## Conclusions

This case report illustrates a rare presentation of RCC in the anterior orbit as the first presenting sign of RCC metastasis after a five-year remission period. Metastatic RCC to the anterior orbit is difficult to clinically diagnose due to its similarity of presentation to benign conditions and other malignant tumors. RCC metastasis to the eye should be in the differential diagnosis of patients presenting with a mass in the eyelid, and biopsy should be performed when the suspicion is supported by history, imaging, and ocular examination. 
